# The healthcare costs of antimicrobial resistance in Lebanon: a multi-centre prospective cohort study from the payer perspective

**DOI:** 10.1186/s12879-021-06084-w

**Published:** 2021-05-01

**Authors:** Katia Iskandar, Christine Roques, Souheil Hallit, Rola Husni-Samaha, Natalia Dirani, Rana Rizk, Rachel Abdo, Yasmina Yared, Matta Matta, Inas Mostafa, Roula Matta, Pascale Salameh, Laurent Molinier

**Affiliations:** 1 Department of Mathématiques Informatique et Télécommunications, Université Toulouse III, Paul Sabatier, INSERM, UMR 1295, F-31000 Toulouse, France; 2 INSPECT-LB: Institut National de Santé Publique, d’Épidémiologie Clinique et de Toxicologie-Liban, Beirut, Lebanon; 3 Department of Pharmacy, Lebanese University, Mount Lebanon, Beirut, Lebanon; 4 Department of Bioprocédés et Systèmes Microbiens, Laboratoire de Génie Chimique, Université Paul Sabatier Toulouse III, UMR 5503, Toulouse, France; 5 Department of Bactériologie-Hygiène, Centre Hospitalier Universitaire, Toulouse, Hôpital Purpan, Toulouse, France; 6 Faculty of Medicine and Medical Sciences, Holy Spirit University of Kaslik (USEK), Jounieh, Lebanon; 7 Department of Medicine, Lebanese American University, Byblos, Lebanon; 8 Department of Infection Control, Lebanese American University Medical Center, Beirut, Lebanon; 9 Department of Infectious Diseases, Dar El Amal University Hospital, Baalbeck, Lebanon; 10 Department of Health Services Research, School CAPHRI, Care and Public Health Research Institute, Maastricht University, 6200 MD Maastricht, The Netherlands; 11 Medical School, University of Nicosia, Nicosia, Cyprus; 12 Department of Clinical Pharmacy, Geitaoui Hospital, Beirut, Lebanon; 13 Department of Medicine, St Joseph University, Beirut, Lebanon; 14 Department of Quality and Safety, Nabatieh Governmental Hospital, Nabatieh, Lebanon; 15 Department of Medical Information, Centre Hospitalier Universitaire, INSERM, UMR 1027, Université Paul Sabatier Toulouse III, F-31000 Toulouse, France

**Keywords:** Antimicrobial resistance, Healthcare cost, Length of stay, In-hospital mortality

## Abstract

**Background:**

Our aim was to examine whether the length of stay, hospital charges and in-hospital mortality attributable to healthcare- and community-associated infections due to antimicrobial-resistant bacteria were higher compared with those due to susceptible bacteria in the Lebanese healthcare settings using different methodology of analysis from the payer perspective .

**Methods:**

We performed a multi-centre prospective cohort study in ten hospitals across Lebanon. The sample size consisted of 1289 patients with documented healthcare-associated infection (HAI) or community-associated infection (CAI). We conducted three separate analysis to adjust for confounders and time-dependent bias: (1) Post-HAIs in which we included the excess LOS and hospital charges incurred after infection and (2) Matched cohort, in which we matched the patients based on propensity score estimates (3) The conventional method, in which we considered the entire hospital stay and allocated charges attributable to CAI. The linear regression models accounted for multiple confounders.

**Results:**

HAIs and CAIs with resistant versus susceptible bacteria were associated with a significant excess length of hospital stay (2.69 days [95% CI,1.5–3.9]; *p* < 0.001) and (2.2 days [95% CI,1.2–3.3]; p < 0.001) and resulted in additional hospital charges ($1807 [95% CI, 1046–2569]; p < 0.001) and ($889 [95% CI, 378–1400]; *p* = 0.001) respectively. Compared with the post-HAIs analysis, the matched cohort method showed a reduction by 26 and 13% in hospital charges and LOS estimates respectively. Infections with resistant bacteria did not decrease the time to in-hospital mortality, for both healthcare- or community-associated infections. Resistant cases in the post-HAIs analysis showed a significantly higher risk of in-hospital mortality (odds ratio, 0.517 [95% CI, 0.327–0.820]; *p* = 0.05).

**Conclusion:**

This is the first nationwide study that quantifies the healthcare costs of antimicrobial resistance in Lebanon. For cases with HAIs, matched cohort analysis showed more conservative estimates compared with post-HAIs method. The differences in estimates highlight the need for a unified methodology to estimate the burden of antimicrobial resistance in order to accurately advise health policy makers and prioritize resources expenditure.

## Background

Antibiotics are known to generate a positive externality on society by preventing the spread of bacterial infections [1–4]. Since their discovery in the past century, the misuse and abuse of antibiotics [5–9] has led to the selection of resistant bacteria and generated extra costs that yielded to a negative societal externality by increasing the burden of bacterial illnesses and eroding the effectiveness of antibiotics for future cases [4–10]. In recent years, antibiotic resistance became a public health priority and triggered global actions. One Health initiatives [11–13] tried to address current related market failures and support measures to mitigate its detrimental worldwide impact [14, 15]. With the increased limitations in healthcare resources, expenditures on programs that reduce resistance must efficiently be allocated [2]. Accurate estimates of the economic burden of antimicrobial resistance (AMR) provide a baseline tool to study the cost-effectiveness of interventions to mitigate this global crisis and gives policymakers the incentives needed to invest in research and funding programs that track and prevent the spread of resistance [10–19]. Literature review in the field shows that health economics failed so far to demonstrate the real cost of resistance [10–20]. Published studies quantifying the economic burden of AMR reported a wide variability of cost estimates and showed heterogeneity of methodological assumptions [10, 18, 21–27]. The majority of data findings originate from high-income countries with unique Health Systems that limit the comparability of results with Low- and Middle-income countries (LMICs) and highlight the gap in research in these regions [28]. Lebanon is a middle-income country facing multiple challenges ranging from scarce financial resources, migrants and asylum seekers challenges, loose health system rules and regulations, economic and ecosystem issues, in addition to the fragmented health system and the diversity of health policy. In a country struggling for cost containment, the estimates of the economic and healthcare burden of AMR needed from multiple perspectives.

Research in the field has mainly focused on the epidemiology of selected pathogens and their virulence [29, 30] and the health impact of resistance [29, 31–50]. Current data on AMR originate from academic research and the society of infectious diseases that both collaborate with different public and private hospitals [29, 30, 46]. The economic impact of AMR in Lebanon may be high compared with other middle-income countries due to the availability of antibiotics over-the-counter [29], the high incidence of AMR [29], the misuse and abuse of antibiotics in human health, animal health and the agricultural sector [51, 52]. Quantifying the burden of AMR in the healthcare settings is the only starting point to estimate the costs of resistance and provide high-quality estimates based on available recommendations from different systematic reviews, published literature, and experts in the field [10, 18, 20, 22, 27]. Here, we aimed to quantify the excess healthcare costs, length of stay, and in-hospital mortality associated with antimicrobial-susceptible versus -resistant infections from the payer perspective using a conventional method for studying the costs of community-associated-infections (CAIs) and two different methodologies of analysis previously adapted in the literature [53, 54] for estimating the costs of healthcare-associated- infections (HAIs) and compare attributable results.

## Context

The republic of Lebanon is a democratic, parliamentary state, classified by the World Bank (2020) as an upper-middle income country [55]. It is located in the near east, at the crossroads of the three continents: Europe, Asia, and Africa. The long history of conflicts contributed to the economic downturn and fragile health system and to the development of non-governmental organizations (NGOs) and private institutions that became the major provider of health services as well as the main contractor to the Ministry of Public Health (MOPH) for the provision of healthcare [56]. The total number of hospitals in Lebanon is 165, among which the private sector accounts for the vast majority of 137 institutions mostly located in the capital region. Hospitals in Lebanon cover acute as well as long-term stays and offer all types of medical and surgical services. The vast majority of public institutions have less than 100 beds and autonomous governance while the total number of hospital beds is 2550 [56]. The private sector is the primary provider of healthcare services in Lebanon. The number of beds varies between 80 and 400 beds. This sector is well equipped with highly trained staff and variable financial resources, offering superior multidisciplinary services. The private sector makes up a total of 12,648 hospital beds.

## Methods

### a. Study design

We performed a prospective multi-centre cross-sectional cohort study in tertiary acute-care centers across Lebanon from the payer perspective. We assumed that infections due to resistant- compared with –susceptible bacteria were the independent exposure of interest, and we evaluated their impact on excess LOS, excess hospital charges, and in-hospital mortality.

### b. Settings

Hospitals were either teaching or non-teaching, from private or public sectors. We contacted hospital directors and Institutional Review Boards (IRB) to grant us access to the hospital financial, clinical, and microbiological database. Hospitals are enrolled based on the location and their willingness to share financial data. The majority were reluctant. Each hospital gave its ethical approval before proceeding to data collection. Patient consent is not obtained as data were collected and analyzed anonymously. The study started in January 2016 and ended in December 2017.

#### Exposures

We considered all documented patients exposures defined by the causative bacteria (gram-positive and gram-negative) and antimicrobial susceptibility testing due to either antibiotic-resistant or –susceptible isolates other than tuberculosis. We excluded (1) the probabilistic infections considered non-documented infections and (2) the cases of colonization defined by the Center of Diseases control and prevention (CDC) [100] as “the presence of microorganisms on skin, on mucous membranes, in open wounds, or in excretions or secretions but are not causing adverse clinical signs or symptoms”. We labeled non-susceptible and intermediate susceptibility as resistant. We considered high priority pathogens according to the definition of the World Health Organization (WHO) and the Centers for Disease Control (CDC), a primary priority of the study [6, 30, 57]. We defined antimicrobial acquired resistance as:

- *Enterobacteriaceae* resistant to carbapenems, fluoroquinolones, aminoglycosides, and third-generation cephalosporins
- *Acinetobacter baumanii* resistant to carbapenems
- *Pseudomonas aeruginosa* resistant to carbapenems
- *Enterococcus faecalis* and *Enterococcus faecium* resistant to vancomycin
- *Staphylococcus aureus* resistant to oxacillin
- *Streptococcus pneumoniae* resistant to penicillins, and *Streptococcus B* resistant to clindamycin

#### Data collection

We recruited five investigators, four on-site in addition to one research assistant, who were all trained on standardized data collection. We adapted the data collection sheet based on similar cohort studies [58–64] and the particularities of the Lebanese healthcare system. Covariates fell into the following three broad categories: (1) patient demographic characteristics at baseline, (2) factors present at admission, (3) factors arising during the hospitalization before the onset of infection, (4) antibiotic susceptibility testing results in addition to (5) the issued hospital charges per patient case (proforma). All events were adequately recorded, including time zero defined as the time of admission, in addition to the time to onset of infection, time of patient enrollment, and time of hospital discharge alive or dead [65–67]^.^

### c. Participants

We enrolled 1289 adult patients hospitalized for more than 48 h with either healthcare- or community-associated infection due to at least one antimicrobial-resistant bacteria or to all antimicrobial –susceptible bacteria (control group). The excluded cases were patients admitted to the ambulatory care unit, to a psychiatric unit, or other non-acute care units. We also excluded infections with *Mycobacterium sp. P*atients with an extended length of stay (LOS) post-infection that exceeded 45 days were not enrolled in the study because we consider based on local evidence that they may be attributable to other conditions like those related to underlying non-communicable diseases. Patients were diagnosed with either CAIs or HAIs associated with one or multiple bacteria. Resistant cases are those infected with at least one resistant bacteria documented by the results of antibiotic susceptibility testing. Patient were followed from the date of admission till discharge (i.e. alive or dead) from the hospital (Fig. 1).

### d. Covariates

We considered as potential confounders the patient characteristics at baseline like patient age, gender, and patient comorbidity [68], and hospitalization(s) within the last 30 days. The dependent variables were excess LOS, excess hospital charges, and time to in-hospital mortality, all measured post-infection. The accurate recording of the date and time stamps made it possible to determine the time-varying factors arising before and after an infection like mechanical ventilation, insertion of a catheter, and stay in the intensive care unit. We did not account for events occurring post-infection to avoid the control of intermediates in the causal pathway. We included the severity of illness represented by the acute physiological assessment and chronic health evaluation (APACHE) score [69] for descriptive purposes only since the timing of score determination was unknown. We accounted for the appropriateness of empirical antibiotic therapy (i.e. the administration of the antibiotics, after the time of specimen culture, to which specific isolate displayed in vitro susceptibility) to avoid the overestimation of the effect of AMR on patient outcomes.

### e. Data sources and measurements

#### Microbiological method

We obtained the antimicrobial susceptibility testing results from the hospital laboratory and microbiology database. All laboratories adhered to current guidelines mainly from the Clinical and Laboratory Standards Institute (CLSI), in addition to the European Committee on Antimicrobial Susceptibility Testing (EUCAST) and the Antibiogram Committee of the French Microbiology Society (CA-SFM) [70, 71].

#### Cost estimation

The sources of costs are the hospital’s financial database. Data included detailed total hospital stay charges, pharmacy expenditures, antibiotics charges, medical accessories, and serums, laboratory and pathology laboratory charges, imaging charges, Mechanical ventilation, third-party payer reimbursement, patient co-payment, and total hospitalization charges. All costs were expressed in Lebanese pounds and converted to US dollar currency (1US dollar =1507.5 Lebanese Lira L.L). Total hospitalization costs may include infection prevention and control costs that were not provided by the hospitals in detail. The cost of hospitalization per patient case is the product of the billed charges during the hospitalization.

Each case is assigned a number generated by the institutional database.

### f. Outcomes of interest

The primary outcomes were to estimate the excess hospital charges, excess LOS, and in-hospital mortality in patients with CAIs and HAIs due to antibiotic-resistant compared with susceptible bacteria from the payer perspective and to compare the differences in the results for cases of HAIs between post-HAIs and matched cohorts analysis.

### g. Statistical analysis

For data entry, we used the Statistical Package for Social Sciences (SPSS) version 23.0 software (SPSS Inc., Chicago, IL, USA). We conducted a separate analysis for community-associated infections and healthcare-associated infections due to antibiotic-resistant versus -susceptible bacterial isolates. For CAIs, we used the conventional method and accounted for the LOS, hospital discharge, and in-hospital mortality for the entire hospital stay from admission to patient discharge from the hospital. We performed two different analyses for cases with HAIs and adjusted for the LOS, hospital charges, and mortality incurred post-infection: (1) Post-infection HAIs where we accounted in terms of hospital charges and LOS for the time from infection as defined by the date of the first culture till discharge from the hospital alive or dead and (2) matched analysis to minimize the risk of time-dependent bias where patients were matched at the analysis stage based on age, gender, Charlson comorbidity index, and on time to infection using the propensity score matching method. We undertook the two analyses to compare the resulting estimates and their effect on outcomes of interest. We also adjusted for the time-varying confounders before infection and accounted for the variable of the timing of infection to decrease the time-dependent bias.

#### Descriptive statistics

We used the student t-test for quantitative variables and the Chi-square test for qualitative variables. We summarized the continuous variables as median with interquartile (IQR), or as means with standard deviation (SD), and the ordinal variables as count with percentage.

#### Estimation of the excess LOS and excess hospital charges

We built linear regression models to identify the impact of independent variables on the excess LOS and excess hospital charges; the independent variable in all models was the infection with resistant bacteria. All regression models assumed a linear relationship between variables and outcomes of interest.

For patients with CAIs, we used the stepwise forward regression analysis procedure. We accounted for baseline characteristics and associated factors arising at admission. Independent variables in the model included age, gender, and comorbidity, in addition to previous exposure to antibiotics.

For HAIs, we build two separate models to compare the effect of independent variables on the outcomes of interest: (1) the Post-HAIs cohort and (2) the matched cohorts. To correct for time variation of some exposures, we included in the models the stay in the intensive care unit and the time to infection as the time-varying factors arising before infection, in addition to other independent variables listed in the CAIs analysis. Other variables like the severity of illness score and the insertion of a catheter were not adjusted for in the model because the timing of events was unknown.

#### Estimation of in-hospital mortality

We used cox proportional hazards models to investigate the association between infections due to antibiotic-resistant versus –susceptible bacteria and the risk of in-hospital mortality. We included the same variables used for the linear regression models above for CAIs and HAIs in addition to the appropriateness of empirical antibiotic therapy. We performed a stepwise analysis. We also built logistic regression models and accounted for the same variables previously included in cox regression using the stepwise analysis to test factors associated with in-hospital mortality in patients with resistant versus susceptible infections.

## Results

The range of bacterial isolates associated with the community- and healthcare-infections are listed as high alerts by the World Health Organization (WHO) and the Center for Diseases Prevention and Control (CDC). The isolated bacteria highly encountered in cases of HAIs compared with CAIs are *Acinetobacter baumannii* carbapenem-resistant, *Pseudomonas aeruginosa* carbapenem-resistant, multidrug resistant (MDR) *Pseudomonas aeruginosa,* and *Methicillin-resistant Staphylococcus aureus (MRSA). Enterobacteriaceae* either *ESBL-producing or* carbapenem-resistant are nearly equally associated with CAIs and HAIs. (Table 1).

**Table 1:** Antibiotic-resistant bacteria associated with Community- and Healthcare associated infections

	Community-associated infection	Healthcare-associated infection
	*n*	*n*
Carbapenem-resistant *Acinetobacter baumannii*	8 (16%)	42 (84%)
Carbapenem-resistant *Pseudomonas aeruginosa*	7 (21%)	26 (79%)
Multidrug-resistant *Pseudomonas aeruginosa*	11 (34%)	21 (66%)
Carbapenem-resistant *Enterobacteriaceae*	10 (43%)	13 (56%)
*Enterobacteriaceae*, *ESBL-producing*	148 (45%)	181 (55%)
*Methicillin-resistant Staphylococcus aureus (MRSA)*	*12 (35%)*	*22 (65%)*
*Erythromycin-resistant group A streptococcus*	5 (71%)	2 (29%)

Data are displayed as number (%) of isolated bacteria

The baseline demographic characteristics of enrolled patient’s show that multiple factors are associated with resistant cases either arising at baseline or during hospitalization. Prior exposure to antibiotics was the only common risk factor for developing either community- or -healthcare-associated infections due to resistant bacteria. A higher Charlson index score was a significant risk for developing CAIs with resistant bacteria. During hospitalization, clinical factors significantly associated with infections due to resistant cases included transfer to the ICU, insertion of a urinary catheter, mechanical ventilation, days of mechanical ventilation, and inappropriateness of antibiotic therapy. Not all listed clinical parameters exhibited significant results in the matched cohort group. CAIs and HAIs associated with resistant bacteria were at a higher risk of all-cause mortality and an increase in the hospital charges estimates. The median LOS post-infection did not significantly differ in matched patients with resistant compared with susceptible cohorts in both groups (CAIs and post-HAIs). (Table 2).

**Table 2:** Patient characteristics, clinical and economic outcomes

	Healthcare-associated infections						Community-associated infections		
	Post-HAI method			Matched Method			Conventional method		
	Resistant	Susceptible	*p*-values	Resistant	Susceptible	*p*-values	Resistant	Susceptible	*p*-values
No.	374	283		238	238		299	333	
**Characteristics prior to infection**									
**Age (median [IQR])**	69 (56–80)	67 (52–78)	0.174	67 (55–80)	68 (53–79)	1.000	81 (69–85)	70 (46–79)	0.152
**Female sex**	170 (45%)	135 (48%)	0.311	105 (44%)	117 (49%)	0.156	152 (51%)	191 (57%)	0.059
**Previous hospitalization**^**a**^	250 (67%)	182 (64%)	0.276	169 (71%)	147 (62%)	0.021	–	–	1.000
**Transfer from another healthcare facility**	22 (6%)	18 (6%)	0.462	15 (6%)	14 (6%)	0.500	–	–	
**Invasive surgery**	124 (33%)	80 (28%)	0.104	74 (31%)	73 (31%)	0.500	66 (22%)	60 (18%)	0.120
**Charlson Co-morbidity Index (median [IQR])**	4 (3–6)	4 (2–6)	0.205	4 (3–6)	4 (2–6)	0.854	6 (5–8)	5 (1–6)	0.002
**Previous antibiotic exposure**^**a**^	67 (18%)	35 (12%)	0.032	46 (19%)	32 (13%)	0.054	52 (17%)	36 (1%)	0.012
**Infection and patient outcomes**									
**A-Clinical parameters**									
**ICU admission**	124 (33%)	75 (26%)	0.040	70 (29%)	60 (25%)	0.177	66 (22%)	39 (12%)	< 0.001
**ICU days (median [IQR])**	9 (4–18)	6 (3–11)	0.144	8 (4–18)	6 (3–10)	0.237	8 (12–19)	6 (2–20)	0.987
**Central venous catheter**	60 (16%)	35 (12%)	0.112	35 (15%)	25 (10%)	0.107	27 (10%)	15 (36%)	0.017
**Central venous catheter days (median [IQR])**	11 (4–20)	8 (4–11)	0.164	12 (5–18)	7 (4–11)	0.076	11 (5–17)	6 (3–20)	0.894
**Urinary catheter**	143 (38%)	90 (32%)	0.052	82 (48%)	89 (37%)	0.283	85 (29%)	61 (18%)	0.004
**Urinary catheter days (median [IQR])**	8 (5–16)	8 (5–11)	0.385	7 (5–16)	8 (5–11)	0.644	12 (8–18)	6 (3.5–19.5)	0.943
**Mechanical ventilation**	86 (23%)	42 (15%)	0.005	48 (20%)	36 (15%)	0.093	29 (10%)	16 (5%)	0.013
**Mechanical ventilation days (median [IQR])**	9 (2.5–17)	0 (0–4)	< 0.001	7 (2–17)	0 (0–4)	0.001	11 (5–15)	1 (0–5)	0.002
**Infection site**									
Bloodstream	65 (56%)	51 (18%)	0.455	44 (18%)	39 (16%)	0.315	39 (13%)	44 (13%)	0.522
Urinary tract	213 (57%)	115 (41%)	< 0.001	134 (56%)	97 (41%)	< 0.001	197 (66%)	201 (60%)	0.053
Respiratory tract	92 (24%)	44 (15%)	0.003	41 (17%)	50 (21%)	0.176	27 (9%)	32 (10%)	0.456
Intraabdominal	16 (4%)	29 (10%)	0.002	12 (5%)	20 (8%)	0.100	17 (6%)	34 (10%)	0.025
Skin/Soft tissue/Osteomyelitis	88 (23%)	76 (27%)	0.188	63 (26%)	71 (30%)	0.238	60 (20%)	41 (12%)	0.002
**Positive culture ≥ 2**	88 (23%)	32 (11%)	< 0.001	59 (25%)	29 (12%)	< 0.001	51 (17%)	257%)	< 0.001
**Polymicrobial**	74 (20%)	19 (7%)	< 0.001	54 (75%)	18 (8%)	< 0.001	44 (15%)	20 (6%)	< 0.001
**APACHE score II (median [IQR])**	6 (1–16)	1 (1–12)	0.527	4 (1–19)	1 (1–12)	0.706	–	–	
**Appropriate antibiotic therapy**	203 (54%)	248 (88%)	< 0.001	203 (85%)	203 (85%)	1.000	193 (64%)	287 (86%)	< 0.001
**In-hospital Mortality**	81 (72%)	32 (28%)	< 0.001	41 (61%)	26 (39%)	0.032	24 (65%)	13 (35%)	0.021
Mortality due to infection	49 (77%)	15 (23%)	0.135	25 (64%)	14 (36%)	0.373	11 (58%)	8 (42%)	0.286
**B-Economic parameters**									
**LOS prior to index date (median [IQR]),days**	0 (0–4)	0 (0–4)	0.426	0 (0–4)	0 (0–4)	0.520	–	–	
**LOS post index date (median [IQR]),days**	7 (4–14)	6 (3–10)	0.016	7 (5–14)	7 (4–11)	0.408	7 (4–12)	5 (3–8)	< 0.001
**Stay charges at ward level**^**b**^	761 (1094)	643 (797)	0.059	790 (1181)	661 (792)	0.200	534 (590)	364 (323)	< 0.001
**ICU stay charges**^**b**^	852 (2049)	508 (1252)	0.007	726 (1930)	466 (1158)	0.077	366 (1093)	240 (47)	0.109
**Total stay charges**^**b**^	1122 (1532)	910 (1350)	0.063	1045 (1402)	894 (1260)	0.220	851 (1151)	578 (822)	0.001
**Pharmaceuticals charges**^**b**^	1872 (3137)	1129 (1680)	< 0.001	1851 (3458)	1030 (1505)	0.001	952 (1291)	628 (1191)	0.001
Antibiotics charges^b^	639 (1365)	314 (598)	< 0.001	609 (1475)	269 (426)	0.001	378 (649)	193 (366)	< 0.001
**Medical Accessories charges**^**b**^	252 (1120)	163 (484)	0.243	231 (1176)	165 (461)	0.425	91 (288)	69 (450)	0.476
**Oxygen charges**^**b**^	135 (658)	52 (151)	0.016	132 (760)	54 (151)	0.121	40 (168)	19 (73)	0.037
**Imaging charges**^**b**^	310 (387)	292 (400)	0.224	318 (425)	277 (390)	0.280	252 (282)	185 (221)	0.001
**Laboratory and microbiology charges**^**b**^	893 (1040)	756 (910)	0.018	868 (984)	714 (872)	0.071	638 (1080)	435 (413)	0.002
**Third-party payment**^**b**^	3865 (4673)	2709 (3053)	< 0.001	3740 (4826)	2567 (2701)	0.001	2829 (3183)	1930 (2186)	< 0.001
**Patient co-payment**^**b**^	379 (1684)	126 (764)	< 0.001	513 (1066)	341 (582)	0.030	263 (1217)	104 (492)	0.001
**Total charges from index date**^**b**^	4363 (5052)	2800 (3221)	< 0.001	4278 (5027)	2882 (3108)	< 0.001	329 (601)	199 (321)	< 0.001

Data are displayed as mean (standard deviation) or number (%) of patients unless indicated otherwise

Percentages were rounded to the nearest whole number

Index date: date of first positive culture

a Within the preceding 90 days

b Hospital charges from index date till discharge expressed as mean (SD), USD

Community-associated infections were defined as those diagnosed within first 3 days of hospitalization in patients who had not been hospitalized in the previous 7 days. Healthcare-associated infections were defined as those diagnosed on third day in hospital or later

Abbreviations: n, sample size; IQR, interquartile range; SD, standard deviation; ICU, Intensive care unit; CV, Central venous; APACHE II, Acute Physiology and Chronic Health Evaluation; LOS, Length of stay; USD, United States dollars

The estimates of the excess length of stay and hospital charges are incurred post-infection per patient case either community-or healthcare-associated infections due to antibiotic-resistant compared with -susceptible bacteria. The linear regression shows that CAIs with resistant bacteria extend the length of hospital stay by a statistically significant estimate of (2.2 [95% *CI*, 1.2–3.3]; *p* < 0.001) days and result in an additional ($889 [95% *CI*, 378–1400]; *p* = 0.001) excess hospital charges compared with susceptible cohorts. HAIs due to resistant bacteria showed a statistically significant higher excess hospital charges ($1297 [95% *CI,* 627–1966]; *p* < 0.001) and longer excess LOS (2.1 days [95% *CI*, 0.9–3.2]; *p* < 0.001). Regression results in the matched cohort group showed more conservative estimates compared with the Post-infection HAIs group by a statistically significant lower difference of 31% in excess LOS and 14% excess hospital charges. Previous exposure to antibiotics and stay in the ICU were the independent variables associated with excess LOS in the post-HAI and matched cohort analysis. Results show that higher comorbidity index scores were associated with increased LOS in the CAIs and post-HAIs analysis compared with the matched cohort analysis. Independent variables in the model associated with excess hospital charges were advanced age, and the uses of antibiotics before admission in the post-HAI analysis, the high Charlson comorbidity index score, and ICU stay in the matched cohort analysis model. (Table 3).

**Table 3:** Regression analyses of excess hospital charges and excess LOS attributable to antibiotic-resistant bacteria

	*Unstandardized beta*	*95% Confidence Interval*		***R***^***2***^	*p-value*
		*Upper*	*Lower*		
**Community-associated infection – Conventional method**					
**Excess LOS post-infection, days**	2.2	1.2	3.3	0.027	< 0.001
**Excess hospital charges, $USD**	889	378	1400	0.046	0.001
**Healthcare-associated infection – “Post-infection HAI” method**					
**Excess LOS post-infection, days**	2.1	0.9	3.2	0.140	< 0.001
**Excess hospital charges, $USD**	1297	627	1966	0.274	< 0.001
**Healthcare-associated infection – Matched method**					
**Excess LOS post-infection, days**	1.6	0.3	2.9	0.135	0.016
**Excess hospital charges, $USD**	1139	471	1807	0.229	0.001

Linear regression models were used assuming linear distribution. Unstandardized beta coefficient, excess LOS post-infection and excess hospital charges displayed in the table are those for the key independent variable: infection due at least one resistant bacteria. The other independent variables included in the community-associated infection regression model were patient age, gender, Charlson co-morbidity index and previous antibiotic exposure. In the healthcare-associated infection analyses, “post HAIs” analysis the other independent variables included in the regression models were patient age, gender, Charlson co-morbidity index, previous exposure to antibiotic, transfer to the ICU, insertion of central venous catheter, insertion of urinary catheter, mechanical ventilation, days of mechanical ventilation, time to infection, appropriateness of antibiotic therapy. *p* value < 0.05 is considered significant

Abbreviations: LOS, Length of stay; Coefficient, unstandardized *Beta*; *CI,* confidence interval; HAIs, healthcare –associated infections; ICU, intensive care unit

The results of logistic regression that identifies factors associated with in-hospital mortality. The independent effect of infections with resistant bacteria on in-hospital mortality was significant in cohorts with HAIs (odds ratio, 0.517 [95% *CI,* 0.327–0.820]; *p* = 0.05). A higher Charlson comorbidity index score increased the odds of in-hospital mortality in all cohort groups. An increase in time to infection was an additional independent factor associated with in-hospital mortality due to HAIs in both the Post-HAIs and matched groups. Cox regression models explore the daily hazard of reaching the endpoint of discharge alive or in-hospital mortality in individuals with CAIs and HAIs. The hazard ratio of infection with resistant bacteria did not appear to be significantly associated with an increased hazard of in-hospital mortality. (Table 4).

**Table 4:** Regression model – In-hospital Mortality

	*Odds ratio*	*95% Confidence Interval*		*p-value*
**Community-associated infection**				
Charlson comorbidity index	1.399	1.230	1.590	< 0.001
**Healthcare-associated infection – “Post-infection HAIs” analysis**				
At least one resistant bacteria	0.517	0.327	0.820	0.050
Charlson comorbidity index	1.144	1.038	1.261	0.007
Age	1.024	1.007	1.041	0.005
Time to infection	1.078	1.040	1.117	< 0.001
**Healthcare-associated infection – Propensity score matching analysis**				
Charlson comorbidity index	1.258	1.140	1.389	< 0.001
Time to infection	1.066	1.022	1.111	0.003

The dependent variable is death or discharge alive. The key dependent variable is CAIs or HAIs with at least one resistant bacteria. Other independent variables included in the community-associated infection regression model were patient age, gender and Charlson co-morbidity index. In the healthcare-associated infection models, “post HAIs” and matched cohorts, the other independent variables were patient age, gender, Charlson co-morbidity index, previous exposure to antibiotics, time to infection, transfer to the Intensive care unit, mechanical ventilation, insertion of a urinary catheter, insertion of a central venous catheter, appropriateness of antibiotic therapy

Abbreviations: CAIs, community-associated infections; HAIs, healthcare –associated infections

## Discussion

Our study is the first in Lebanon to quantify nationwide the economic burden of hospitalized cases with CAIs or HAIs from the payer perspective. We compared infections due to antimicrobial-resistant with -susceptible bacteria rather than with uninfected as a control group to avoid overestimation of the economic burden of resistance [23, 24, 72–75]. Although the payer perspective is considered narrow in scope, it allows to demonstrate the differences in outcomes estimates between resistant versus susceptible cases and offer baseline data for policymakers to evaluate the cost-effectiveness of current infection control and prevention practices to prevent AMR, in an era where the health system is struggling with scarce resources [10, 18, 27, 76].

The estimation of costs followed the bottom-up microcosting, considered a reliable method for identifying and valuing relevant cost components in the healthcare setting [17, 77]. The time and date stamp allowed to adjust for multiple confounders arising before the onset of infection and to avoid the allocation of intermediate mediators in the causal pathway [23, 27, 53, 78–80].We only considered the severity of illness represented by the APACHE score in the descriptive analysis because the timing of the score estimation was unknown. Accounting for the severity of illness score is thought to accurately predict the clinical outcomes associated with resistance if the optimal time to estimate the severity of illness is 24 h before the first culture-positive specimen sample [23, 79–82]. We adjusted for the time-dependent bias by matching cohorts based on the day of infection [27, 67, 69].

We used the matched cohort with HAIs methodology at the study analysis stage according to age, gender, comorbidity, and timing of infection [23, 27, 53, 79, 83, 84]. The propensity score matching potentially reduces residual confounding compared with paired cohorts that control for a limited number of variables [10, 85].A published study by Nelson and colleagues (2015) [53] compared three methodologies of analysis: the conventional method, matched cohort analysis that accounted for the timing of infection, and the post-HAI method. Results showed more conservative estimates from the matched cohort analysis compared with the methodology based on post-infection data [53]. Our study showed a 26% reduction in excess hospital charges and a 13% decrease in excess LOS in the matched cohort analysis compared with the post-infection analysis.

Similar to a study by Neidell et al. [54], the comparison of LOS and costs due to resistant versus susceptible infections in healthcare settings showed significantly higher estimates associated with HAIs compared with CAIs. Healthcare- and community-associated infections due to susceptible versus resistant bacteria were associated with significantly higher median excess LOS (2.69 days versus 2.2 days) and mean excess hospital charges ($1807 versus $889). We used the survival analysis to estimate the hazard of in-hospital mortality attributable to resistant cases of infection. The results showed no significant increase in hazard ratio according to the three methods of analysis. The limited sample size associated with death cases during hospitalization and the lack of patient follow-up for at least 30 days post-discharge may potentially be contributory factors.

Systematic reviews in the field [10, 18, 23–27] showed a wide variability of estimates and heterogeneity of the methodology quantifying the burden of AMR. Failure to account for the HAIs as a time-varying exposure and to adjust for multiple confounders, competing risks, and for the time-varying confounders generate erroneous results [10, 18, 23, 27]. The multistate model is a recommended methodology of healthcare costs analysis associated with AMR [18, 27]. This method accounts for time-dependent bias and competing risks but do not adjust for time-varying confounding [18, 27, 86]. The g-formulae may offer more reliable results by accounting for the time-varying confounders in addition to time-dependent bias and competing risks [10, 87, 88]. The g-formula estimates joint causal effects in the presence of a time-varying exposure and confounding. The g-formulae relies on assumptions that should be carefully determined to allow the robustness of the results [89]. Matching is a proposed method to avoid the time-dependent bias if the timing of infection was considered [21, 25]. Multiple studies used this method [55] and matched for patient characteristics like age, and gender or used diagnostic code, the severity of illness, and or the LOS before infection [10]. This reliability of these characteristics as an indicator of AMR infections is arguable not adequately tested [10, 85]. Each methodology of analysis and study design influences the quality of the studies despite that each has pitfalls and limitations [10, 18, 27, 87, 90] Jit et al. 2019 [10] proposed a conceptual framework for quantifying the economic burden of AMR and postulated that obtaining accurate estimates requires rigor and innovation in using the existing methodologies.

A standardized method for study design and analysis is highly needed. This method must take into consideration cross-borders differences and limitations to allow comparability and generalizability of results. Quantifying the burden of resistance may influence the fight against AMR if it provides evidence-based estimates [10, 18, 23, 27]. The global economic downturn has a profound impact on population health and the sustainability of healthcare systems leading to multiple cuts on the budget in both the public and private sectors [91]. Re-allocation of resources and health policy rely on quality data to orientate health expenditures. Data findings may allow us to examine the cost-effectiveness of current practices undertaken to mitigate resistance and orientate for the efficient prioritization of resource allocation to enhance global health [10]. The healthcare costs represent part of the burden of AMR to society. The opportunity cost associated with resistance shows what is missing due to resistance to enhance the welfare of the community if the payer is a public entity or to improve coverage of other diseases if the payer is a private entity. In Lebanon, implemented health policy to tackle AMR needs to strengthen the population-based surveillance on AMR consider the pre-requisite to study the economic burden of resistance [21] and expand to cover not only the human health but also the animal health and agriculture sector interfaces.

The limitations of the study are the factors that may affect the accuracy of the data. Our study included a wide range of infections due to different gram-positive and gram-negative bacteria. Targeting either CAI or HAI type due to an antimicrobial-resistant compared with the same -susceptible species can generate more reliable estimates attributable to the associated outcomes. The same remark applied when targeting one specific mechanism of acquired resistance like carbapenemase production. The methodology of antimicrobial susceptibility testing is another concern due to the lack of standardization throughout the enrolled hospitals or at least no homogeneous methods and breakpoints taken into account to define the resistance. For cost estimation, we followed the bottom-up microcosting from the payer perspective in terms of hospital charges that may vary depending on the health system coverage and third-party contracts. For further accuracy, studies taking into account these limitations are recommended to confirm our findings: the standardization of study design and analysis methodology would allow the comparability and generalizability of results for a more informed national and global action to fight AMR.

## Conclusion

Our study is the first nationwide study that quantifies the economic burden of antimicrobial resistance (AMR) in Lebanese hospitals, in comparison with sensitive bacteria. Matched cohort analysis showed more conservative estimates in cases of HAIs. The differences in estimates highlight the need for a unified methodology to estimate the burden of AMR and accurately advise health policymakers and prioritize resource expenditure on infection prevention and control programs.

## Data Availability

The datasets generated and analyzed during the current study are available in the INSPECT-LB repository, https://inspect-lb.org/data-repository/

## Abbreviations

AMR: Antimicrobial resistance
APACHE: Acute physiological assessment and chronic health evaluation
CA-SFM: Antibiogram Committee of the French Microbiology Society
CAIs: Community-associated infections
CDC: Center of Diseases control and prevention
CLSI: Clinical and Laboratory Standards Institute
EUCAST: European Committee on Antimicrobial Susceptibility Testing
HAIs: Healthcare-associated infections
IRB: Institutional Review Boards
IQR: Interquartile
LMICs: Low- and Middle-income countries
LOS: Length of stay
MDR: Multi-drug resistant
MOPH: Ministry of Public Health
NGOs: Non-governmental organizations
SD: Standard deviation
SPSS: Statistical Package for Social Sciences
WHO: World Health Organization
